# Supplementary material for: The Shape of Success: A Scoping Review of Somatotype in Modern Elite Athletes Across Various Sports
Source: Sports (Basel). 2025 Feb 4;13(2):38. doi: 10.3390/sports13020038 (PMC11860359; doi:10.3390/sports13020038)

Figure S1. Somatoplots of male elite athletes

Figure a) Team sports.

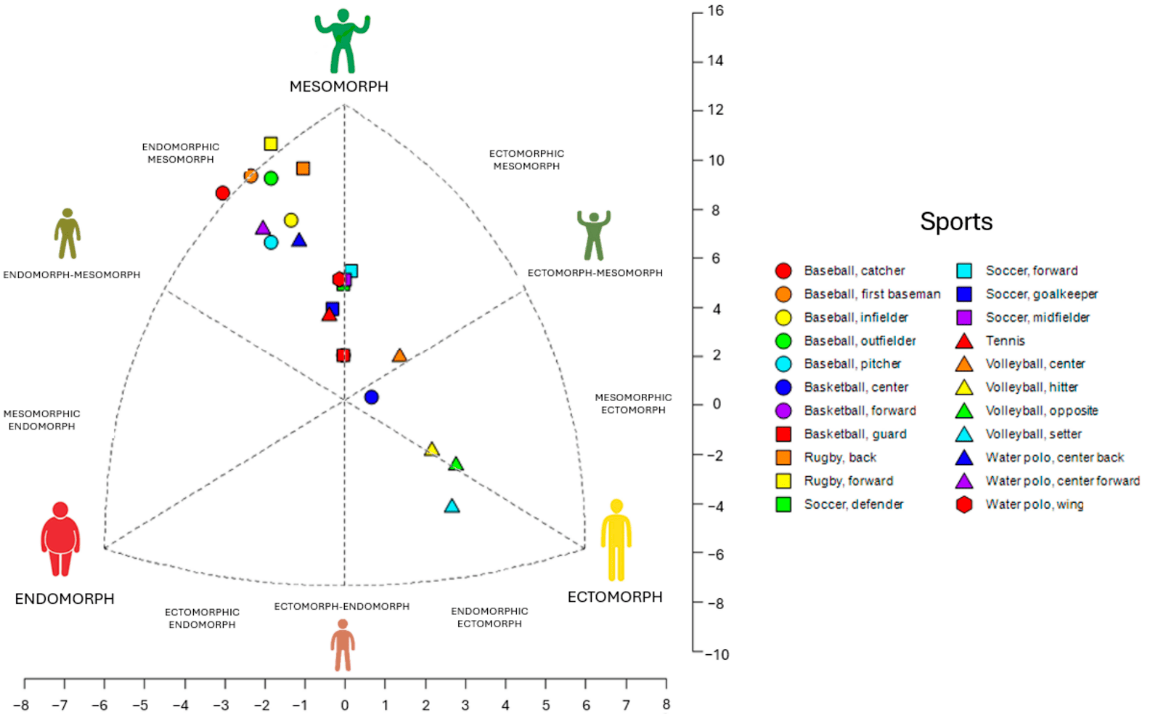

Figure b) Combat sports

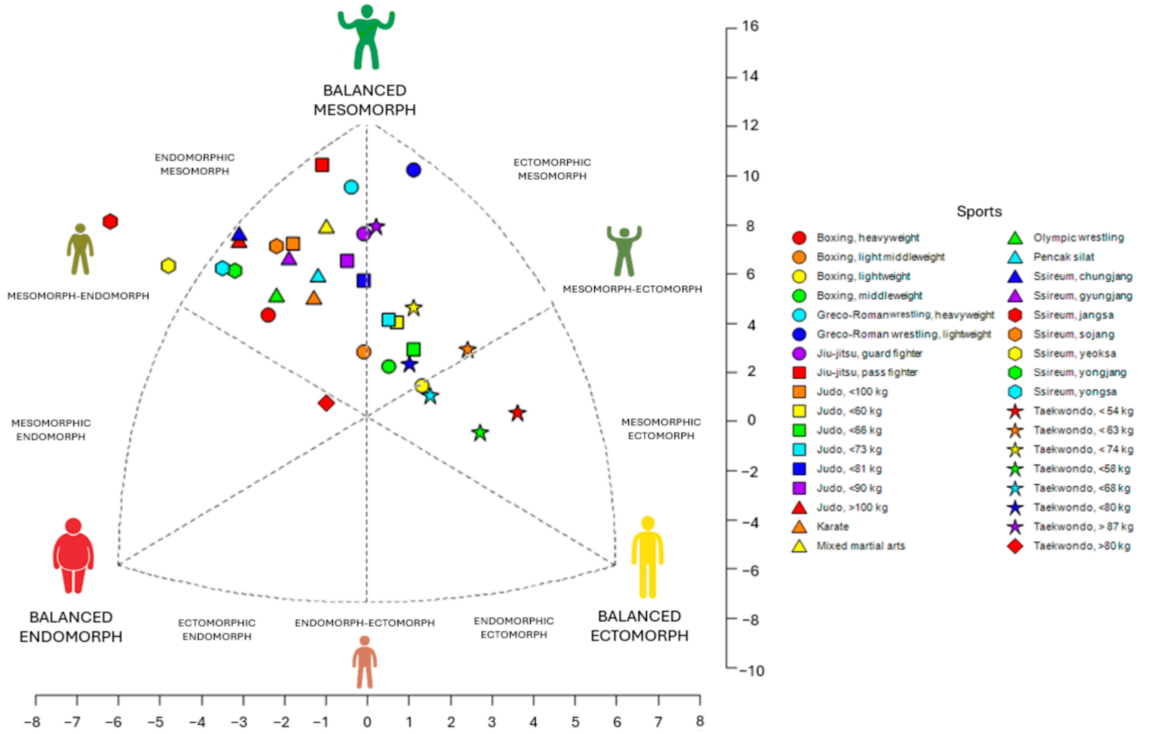

Figure c) Speed and endurance sports.

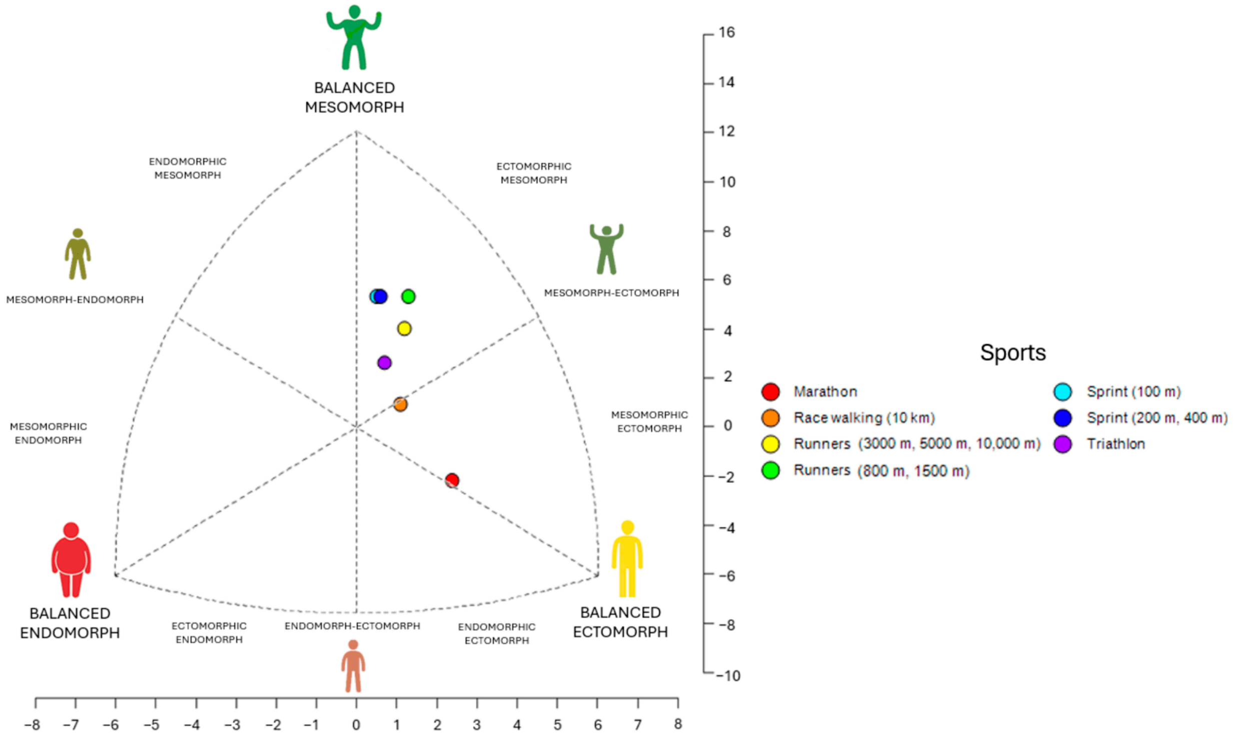

Figure d) Individual sports.

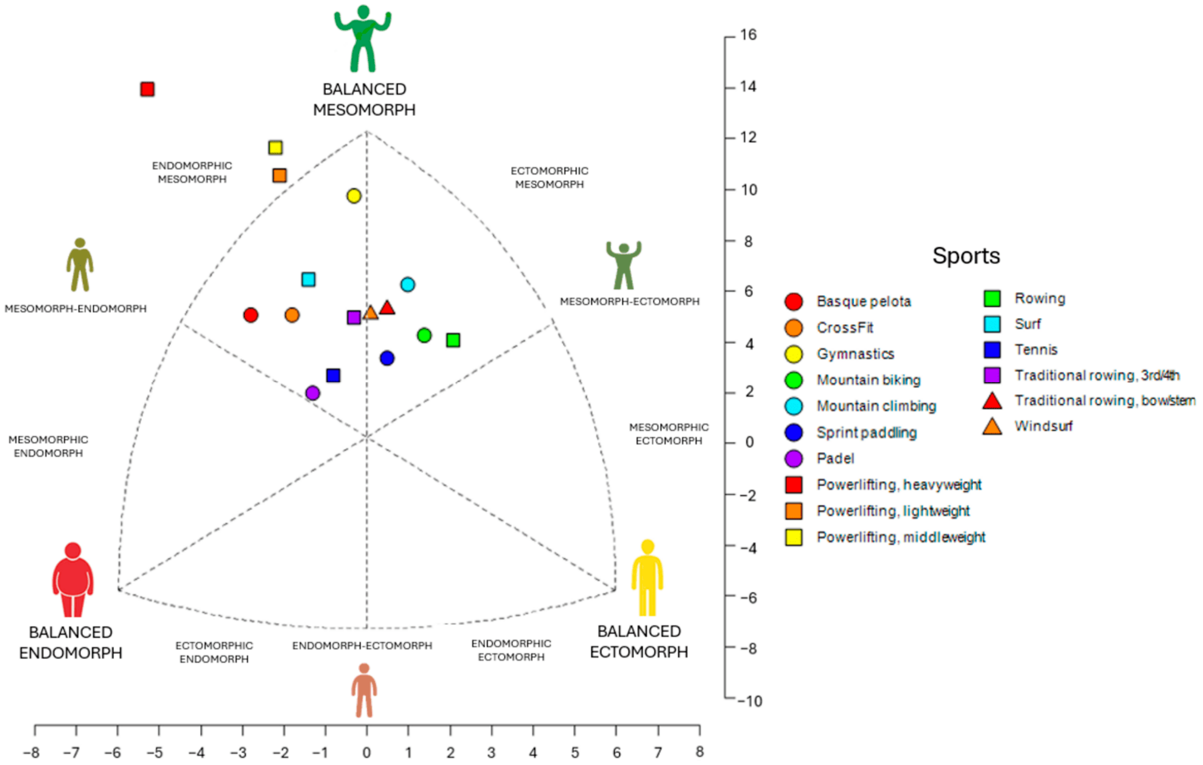

Supplement: Supplementary file 1 [file sports-13-00038-s001.zip › Somatotype Figure S1_male athletes.pdf]
