# Supplementary material for: The Shape of Success: A Scoping Review of Somatotype in Modern Elite Athletes Across Various Sports
Source: Sports (Basel). 2025 Feb 4;13(2):38. doi: 10.3390/sports13020038 (PMC11860359; doi:10.3390/sports13020038)

Figure S3. Somatoplot from different elite levels in judokas

Figure S3a. Somatoplot from male elite judokas

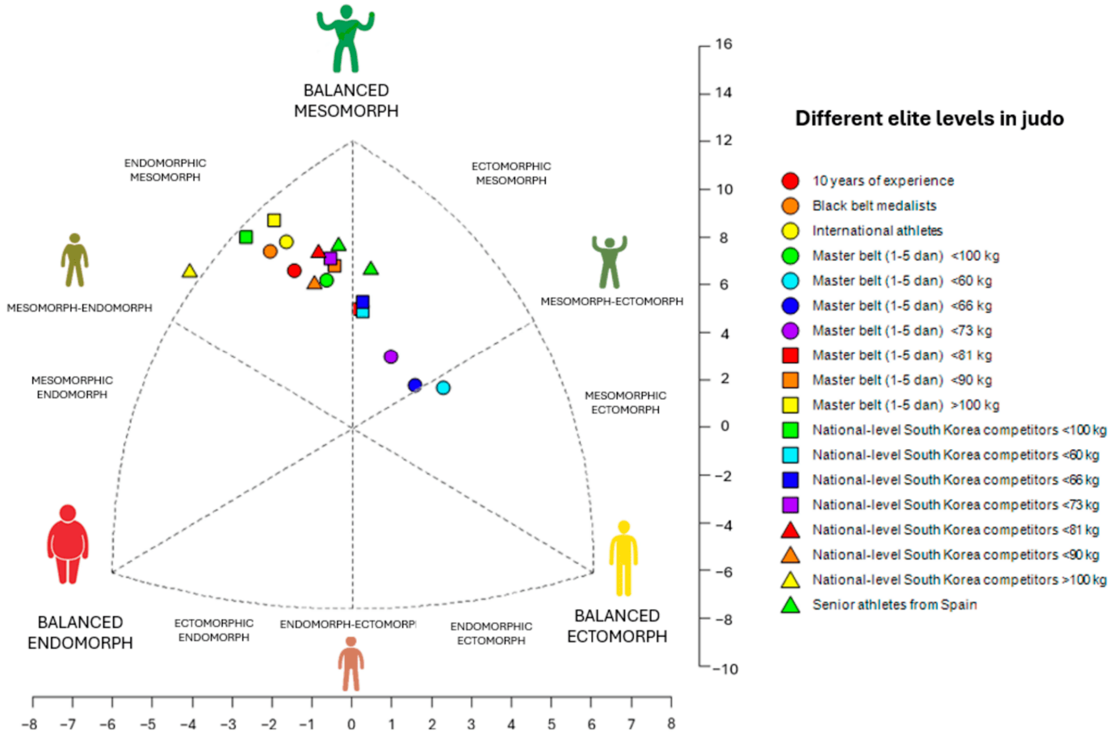

Figure S3b. Somatoplot from female elite judoka

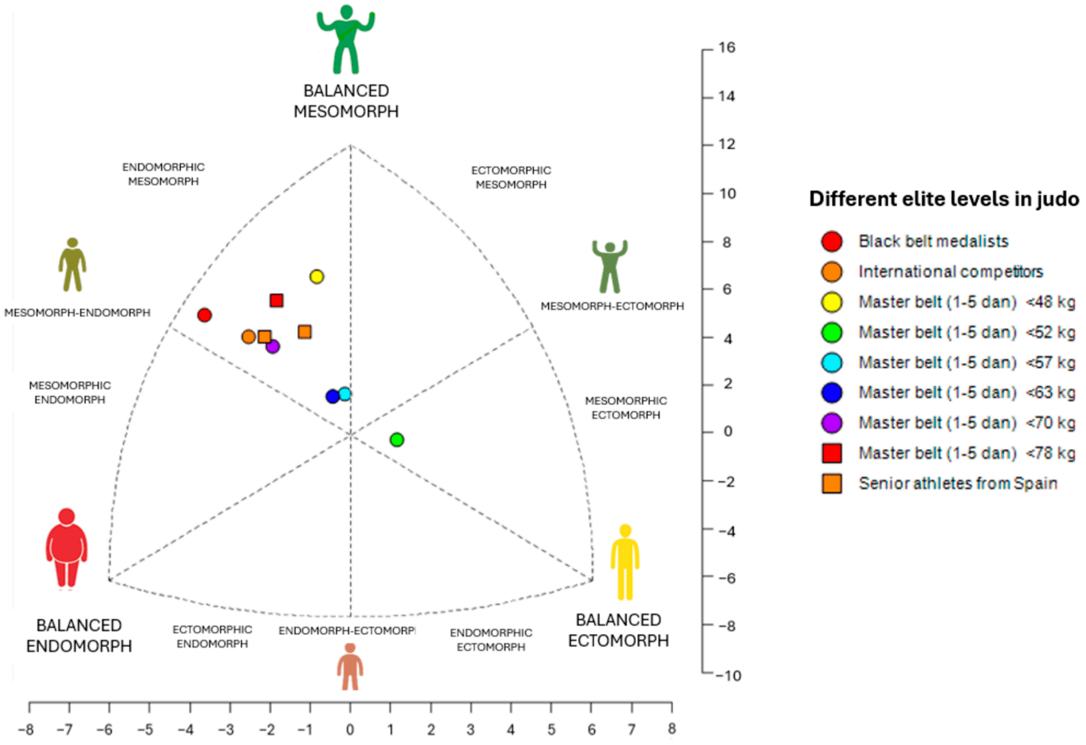

Supplement: Supplementary file 1 [file sports-13-00038-s001.zip › Somatotype Figure S3_judokas.pdf]
