# Supplementary material for: The Shape of Success: A Scoping Review of Somatotype in Modern Elite Athletes Across Various Sports
Source: Sports (Basel). 2025 Feb 4;13(2):38. doi: 10.3390/sports13020038 (PMC11860359; doi:10.3390/sports13020038)
Supplement: Supplementary file 1 [file sports-13-00038-s001.zip › Somatotype Table S2_all athletes.pdf]

Table S2. Characteristics of the sources of evidence from modern elite athletes

Sport	Reference	*n*	Study design	Caliper	Sex	Age	Body mass (kg)	Height (cm)	BMI (kg/m²)	ENDO	MESO	ECTO	Clasification
Bodybuilding, TOP 10 of the South Australian Bodybuilders' Association 1998 and Australian Natural Bodybuilding Organization 1999	van der Ploeg et al., (2001) [55]	5	Case-control	Harpenden	Female	35.60	60.6	167.4	21.2	1.7	4.8	2.7	Ectomorphic mesomorph
Basketball, European Junior Basketball Championship 2000<br><br>Position: forward	Jeličić et al., (2002) [56]	54	Cross-sectional	Not specified	Male	17.78	90.4	198.0	23.0	2.8	3.7	3.8	Central
Basketball, European Junior Basketball Championship 2000<br><br>Position: guard	Jeličić et al., (2002) [56]	53	Cross-sectional	Not specified	Male	17.80	81.3	188.0	23.0	3.2	4.2	3.2	Central
Basketball, European Junior Basketball Championship 2000<br><br>Position: center	Jeličić et al., (2002) [56]	25	Cross-sectional	Not specified	Male	17.80	101.2	205.3	24.0	3	3.5	3.7	Central

Soccer, high level in Turkey	Can et al., (2004) [57]	17	Cross-sectional	Lange	Female	20.73	56.6	162.4	21.5	3.1	3.6	2.4	Mesomorph-endomorph
Rowing, Australian Championships 2003. U-23<br><br>Category: light weight	Slater et al., (2005) [58]	28	Cross-sectional	Harpenden	Female	23.00	57.4	170.0	19.9	2.5	3.3	3.7	Mesomorph-ectomorph
Rowing, Australian Championships 2003. U-23<br><br>Category: light weight	Slater et al., (2005) [58]	17	Cross-sectional	Harpenden	Female	OPEN	57.90	170.3	20.0	2.4	3.3	3.7	Mesomorph-ectomorph
Rowing, Australian Championships 2003. U-23<br><br>Category: light weight	Slater et al., (2005) [58]	35	Cross-sectional	Harpenden	Male	23.00	70.6	181.6	21.4	1.4	4.4	3.6	Ectomorphic mesomorph
Rowing, Australian Championships 2003. U-23<br><br>Category: light weight	Slater et al., (2005) [58]	27	Cross-sectional	Harpenden	Male	OPEN	71.20	180.7	21.2	1.4	4.8	3.4	Ectomorphic mesomorph
Volleyball, England men's junior volleyball squad	Duncan et al., (2006) [59]	Not specified	Cross-sectional	Holtain	Male	17.50	71.2	191.0	19.5	2.6	1.9	5.3	Endomorphic ectomorph

Position: setter													
Volleyball, England men's junior volleyball squad<br><br>Position: opposite	Duncan et al., (2006) [59]	Not specified	Cross-sectional	Holtain	Male	17.50	71.3	190.0	19.8	2.3	2.5	5.1	Balanced ectomorph
Volleyball, England men's junior volleyball squad<br><br>Position: center	Duncan et al., (2006) [59]	Not specified	Cross-sectional	Holtain	Male	17.50	77.6	187.0	22.2	2.2	3.9	3.6	Mesomorph-ectomorph
Volleyball, England men's junior volleyball squad<br><br>Position: hitter	Duncan et al., (2006) [59]	Not specified	Cross-sectional	Holtain	Male	17.50	77.9	193.0	20.9	2.4	2.6	4.6	Balanced ectomorph
Handball, Greek first National League	Bayios et al., (2006) [60]	222	Cross-sectional	John Bull	Female	21.50	65.1	165.9	23.6	4.2	4.7	1.8	Mesomorph-endomorph
Basketball, Greek first National League	Bayios et al., (2006) [60]	133	Cross-sectional	John Bull	Female	22.10	71.5	174.7	23.4	3.7	3.2	2.4	Mesomorph-endomorph
Volleyball, Greek first National League	Bayios et al., (2006) [60]	163	Cross-sectional	John Bull	Female	23.80	69.5	177.1	22.1	3.4	2.7	2.9	Central
Rugby, Australian Rugby League	Lundy et al., (2006) [61]	31	Cross-sectional	Harpenden	Male	25.00	85.5	177.9	27.0	2.2	6.5	1.2	Endomorphic mesomorph

Position: back													
Rugby, Australian Rugby League<br><br>Position: forward	Lundy et al., (2006) [61]	45	Cross-sectional	Harpenden	Male	25.40	98.4	182.7	29.5	2.8	7.2	0.8	Endomorphic mesomorph
Gymnastics, Argentina's National Team	Rodriguez & Berral, (2006) [62]	25	Cross-sectional	Harpenden	Male	21.2	65.5	166.2	23.7	1.9	6.7	1.6	Balanced mesomorph
Tennis, Davis Junior Cup and Fed Junior 2005 y 2006<br><br>Speciality: Doubles	Sánchez-Muñoz et al., (2007) [63]	12	Cross-sectional	Holtain	Male	16.40	70.4	176.9	22.5	2.2	5.3	2.8	Ectomorphic mesomorph
Powerlifting, national and international competitions from New Zealand<br><br>Category: heavyweight	Keogh et al., (2007) [64]	15	Cross-sectional	Slim Guide	Male	33.40	121.9	174.7	39.9	6.3	10.7	0.1	Endomorphic mesomorph
Powerlifting, national and international competitions from New Zealand	Keogh et al., (2007) [64]	9	Cross-sectional	Slim Guide	Male	35.40	68.9	163.0	25.9	3.2	7.5	1.1	Endomorphic mesomorph

Category: lightweight													
Powerlifting, national and international competitions from New Zealand<br><br>Category: middleweight	Keogh et al., (2007) [64]	30	Cross-sectional	Slim Guide	Male	37.90	87.7	174.7	28.7	3.2	8.0	0.7	Endomorphic mesomorph
Volleyball from the Greek National League<br><br>Position: hitter	Malousaris et al., (2008) [65]	19	Cross-sectional	Not specified	Female	25.70	72.80	181.2	22.2	3.3	2.5	3.3	Central
Volleyball from the Greek National League<br><br>Position: center	Malousaris et al., (2008) [65]	26	Cross-sectional	Not specified	Female	25.70	74.30	182	22.4	3.2	2.2	3.2	Central
Volleyball from the Greek National League<br><br>Position: opposite	Malousaris et al., (2008) [65]	8	Cross-sectional	Not specified	Female	25.70	71.40	183.6	21.3	2.6	2.3	3.9	Balanced ectomorph
Volleyball from the Greek National League<br><br>Position: setter	Malousaris et al., (2008) [65]	17	Cross-sectional	Not specified	Female	25.70	67.80	176.9	21.8	3.4	2.2	3.2	Endomorph ectomorph

Volleyball from the Greek National League<br><br>Position: libero	Malousaris et al., (2008) [65]	9	Cross-sectional	No especifica	Female	25.70	63.30	171	21.6	3.2	3.3	2.8	Central
Karate, Philippines' national team	Pieter & Bercades, (2009) [66]	5	Cross-sectional	Lange	Female	20.25	55.8	161	21.5	3.1	3.7	2.4	Endomorphic mesomorph
Fencing, Philippines' national team	Pieter & Bercades, (2009) [66]	6	Cross-sectional	Lange	Female	24.33	54.3	153.3	23.1	3.7	4.8	1.3	Endomorphic mesomorph
Pencak silat, Philippines' national team	Pieter & Bercades, (2009) [66]	5	Cross-sectional	Lange	Female	20.4	53.3	156.3	21.8	4	4.1	1.9	Mesomorph-endomorph
Karate, Philippines' national team	Pieter & Bercades, (2009) [66]	12	Cross-sectional	Lange	Male	24	64.3	169.7	22.3	2.4	4.7	2.6	Balanced mesomorph
Pencak silat, Philippines' national team	Pieter & Bercades, (2009) [66]	8	Cross-sectional	Lange	Male	26.88	70.9	169.9	24.5	3.2	5.6	2	Endomorphic mesomorph
Baseball, 43rd Cuban National Baseball Series<br><br>Position: first baseman	Carvajal et al., (2009) [67]	20	Cross-sectional	Not specified	Male	28.56	98.3	183.7	29.3	3.38	6.83	0.6	Endomorphic mesomorph
Baseball, 43rd Cuban National Baseball Series<br><br>Position: infielder	Carvajal et al., (2009) [67]	26	Cross-sectional	Not specified	Male	28.56	82.7	179	25.8	2.86	5.91	1.54	Endomorphic mesomorph
Baseball, 43rd Cuban National Baseball Series	Carvajal et al., (2009) [67]	20	Cross-sectional	Not specified	Male	28.56	90.16	179.2	28.1	4.08	6.84	0.32	Endomorphic mesomorph

Position: catcher													
Baseball, 43rd Cuban National Baseball Series<br><br>Position: outfielder	Carvajal et al., (2009) [67]	20	Cross-sectional	Not specified	Male	28.56	88.3	177.1	28.2	2.96	6.66	0.66	Endomorphic mesomorph
Baseball, 43rd Cuban National Baseball Series<br><br>Position: pitcher	Carvajal et al., (2009) [67]	20	Cross-sectional	Not specified	Male	28.56	87.6	181.5	26.7	3.26	5.66	1.47	Endomorphic mesomorph
Windsurf, Formula Windsurfing World Championships 2007	Cortell-Tormo et al., (2010) [68]	15	Cross-sectional	Holtain	Male	25.40	83.1	184.6	24.3	2.3	5.0	2.4	Balanced mesomorph
Soccer, Turkey's Super League<br><br>Position: defender	Hazir (2010) [69]	90	Cross-sectional	Holtain	Male	25.9	75.6	178.6	23.7	2.4	4.8	2.3	Balanced mesomorph
Soccer, Turkey's Super League<br><br>Position: forward	Hazir (2010) [69]	56	Cross-sectional	Holtain	Male	25.2	76.6	177.9	24.2	2.4	5	2.1	Balanced mesomorph
Soccer, Turkey's Super League<br><br>Position: midfielder	Hazir (2010) [69]	120	Cross-sectional	Holtain	Male	25.8	73.9	176.1	23.8	2.5	4.9	2.2	Balanced mesomorph

Soccer, Turkey's Super League<br><br>Position: goalkeeper	Hazir (2010) [69]	37	Cross-sectional	Holtain	Male	25.7	82	184.8	24.0	2.9	4.6	2.6	Balanced mesomorph
Judo, Spanish National Team	Franchini et al., (2011) [70]	8	Cross-sectional	Harpenden	Male	22.10	82.5	179.8	25.5	2.1	5.2	1.6	Balanced mesomorph
Judo, Spanish National Team	Franchini et al., (2011) [70]	18	Cross-sectional	Harpenden	Female	24.10	64.7	163.3	24.2	3.8	4.8	1.7	Endomorphic mesomorph
Karate, Polish International Team	Sterkowicz-Przybycień, (2010) [71]	14	Cross-sectional	Holtain	Male	26.70	86.10	179	26.9	3.7	5.8	1.3	Endomorphic mesomorph
Karate, Polish National Team	Sterkowicz-Przybycień, (2010) [71]	16	Cross-sectional	Holtain	Male	23.50	81.40	180	25.1	3.5	5	2	Endomorphic mesomorph
Sprint paddlers, Royal Spanish Canoeing Federation	Alacid et al., (2011) [72]	31	Cross-sectional	Harpenden	Female	14.11	56.27	164.86	20.9	3.6	3.7	3	Central
Sprint paddlers, Royal Spanish Canoeing Federation	Alacid et al., (2011) [72]	64	Cross-sectional	Harpenden	Male	14.16	60.44	169.12	21.1	2.6	4.6	3.1	Balanced mesomorph
Water polo, Spanish Team<br><br>Position: wing	Ferragut et al., (2011) [73]	9	Cross-sectional	Holtain	Male	25.10	82.00	184.4	24.2	2.53	5.01	2.4	Balanced mesomorph
Water polo, Spanish Team<br><br>Position: center back	Ferragut et al., (2011) [73]	5	Cross-sectional	Holtain	Male	21.00	91.40	186.7	26.4	3	5.8	1.9	Endomorphic mesomorph

Water polo, Spanish Team<br><br>Position: center forward	Ferragut et al., (2011) [73]	5	Cross-sectional	Holtain	Male	24.80	102.30	192.1	27.7	3.54	6.14	1.56	Endomorphic mesomorph
Greco-roman wrestling, Polish Team<br><br>Category: heavyweight	Sterkowicz-Przybycień et al., (2011) [74]	12	Cross-sectional	Holtain	Male	23.50	92.40	182	27.9	2.2	6.8	1.1	Endomorphic mesomorph
Greco-roman wrestling, Polish Team<br><br>Category: lightweight	Sterkowicz-Przybycień et al., (2011) [74]	11	Cross-sectional	Holtain	Male	26.50	70.10	168	24.8	1.7	6.3	1.3	Balanced mesomorph
Judo, Judo European Championships Düsseldorf 2003	Sterkowicz-Przybycień & Almansba, (2011) [75]	22	Cross-sectional	Holtain	Male	22.20	87.40	179.6	27.3	3.2	6.32	1.64	Endomorphic mesomorph
Judo, European Judo Championships Bucharest 2004	Sterkowicz-Przybycień & Almansba, (2011) [75]	12	Cross-sectional	Holtain	Female	23.10	74.80	169.3	26.2	4.04	4.89	1.55	Endomorphic mesomorph
Mountain climbing, 21 years of experience	Barbieri et al., (2012) [76]	10	Cross-sectional	Lange	Male	41.40	70.6	176.1	22.7	1.6	5.3	2.6	Ectomorphic mesomorph
Volleyball, Cuban women’s volleyball team<br><br>Position: center	Carvajal et al., (2012) [77]	9	Cross-sectional	Holtain	Female	21.8	79	187.1	22.6	2.9	3.4	3.4	Central

Volleyball, Cuban women's volleyball team<br><br>Position: setter	Carvajal et al., (2012) [77]	8	Cross-sectional	Holtain	Female	23.6	73.7	178.5	23.2	2.6	3.7	2.6	Balanced mesomorph
Volleyball, Cuban women's volleyball team<br><br>Position: hitter	Carvajal et al., (2012) [77]	24	Cross-sectional	Holtain	Female	23.2	74.5	180.7	22.3	2.8	3.6	2.9	Central
Volleyball, highest Spanish league<br><br>Position: setter	Martín-Matillas et al., (2014) [78]	29	Cross-sectional	Holtain	Female	24.80	68.30	176.6	22.0	2.6	3.6	3.1	Central
Volleyball, highest Spanish league<br><br>Position: opposite	Martín-Matillas et al., (2014) [78]	18	Cross-sectional	Holtain	Female	24.80	81.80	183.8	24.4	3.3	3.9	2.5	Endomorphic mesomorph
Volleyball, highest Spanish league<br><br>Position: center	Martín-Matillas et al., (2014) [78]	47	Cross-sectional	Holtain	Female	24.80	75.90	184.8	22.4	2.8	3.2	3.4	Central
Volleyball, highest Spanish league<br><br>Position: hitter	Martín-Matillas et al., (2014) [78]	41	Cross-sectional	Holtain	Female	24.80	76.70	183.6	22.9	2.8	3.4	3.1	Central

Volleyball, highest Spanish league<br><br>Position: libero	Martín-Matillas et al., (2014) [78]	13	Cross-sectional	Holtain	Female	24.80	68.90	171.8	23.5	3.4	4.5	2.1	Endomorphic mesomorph
Marathon, high level from Kenia	Vernillo et al., (2013) [79]	14	Cross-sectional	Holtain	Male	27.71	57.7	171.2	19.7	1.5	1.6	3.9	Balanced ectomorph
Triathlon, 1997 Triathlon World Championships	Landers et al., (2013) [80]	20	Cross-sectional	Not specified	Male	27.5	72.3	179.8	22.6	1.9	4.2	3	Ectomorphic mesomorph
Triathlon, 1997 Triathlon World Championships	Landers et al., (2013) [80]	18	Cross-sectional	Not specified	Female	29.3	59.5	168.3	21.1	2.8	3.6	3	Central
Ssireum wrestling, elite and professional<br><br>Category: gyungjang	Noh et al., (2013a) [81]	7	Cross-sectional	Jamar	Male	21.30	78.90	174.1	26.0	3.2	5.6	1.3	Endomorphic mesomorph
Ssireum wrestling, elite and professional<br><br>Category: sojang	Noh et al., (2013a) [81]	4	Cross-sectional	Jamar	Male	21.30	82.20	174.8	27.1	3.2	5.7	1	Endomorphic mesomorph
Ssireum wrestling, elite and professional<br><br>Category: chungjang	Noh et al., (2013a) [81]	5	Cross-sectional	Jamar	Male	21.30	87.90	176	28.4	4.1	6.4	0.7	Endomorphic mesomorph
Ssireum wrestling, elite and professional	Noh et al., (2013a) [81]	3	Cross-sectional	Jamar	Male	21.30	90.50	177	28.8	4.2	5.7	0.6	Endomorphic mesomorph

Category: yongjang													
Ssireum wrestling, elite and professional<br><br>Category: yongsa	Noh et al., (2013a) [81]	4	Cross-sectional	Jamar	Male	21.30	94.50	179.8	29.5	4.5	5.9	0.6	Endomorphic mesomorph
Ssireum wrestling, elite and professional<br><br>Category: yeoksa	Noh et al., (2013a) [81]	4	Cross-sectional	Jamar	Male	21.30	111.90	182.5	33.8	5.8	6.6	0.2	Endomorphic mesomorph
Ssireum wrestling, elite and professional<br><br>Category: jangsa	Noh et al., (2013a) [81]	5	Cross-sectional	Jamar	Male	21.30	136.30	184.6	40.2	7.2	8.2	0.1	Endomorphic mesomorph
Taekwondo Gyorugi<br><br>Category: < 58 kg	Noh et al., (2013b) [82]	7	Cross-sectional	Jamar	Male	19.70	61.60	174.1	20.3	2.1	2.7	3.9	Mesomorphic ectomorph
Taekwondo Gyorugi<br><br>Category: < 68 kg	Noh et al., (2013b) [82]	12	Cross-sectional	Jamar	Male	19.70	70.70	180.8	21.8	2.3	2.3	3.4	Balanced ectomorph
Taekwondo Gyorugi<br><br>Category: < 80 kg	Noh et al., (2013b) [82]	9	Cross-sectional	Jamar	Male	19.70	77.20	183.1	23.0	1.9	3.1	3.1	Mesomorph-ectomorph
Taekwondo Gyorugi<br><br>Category: > 80 kg	Noh et al., (2013b) [82]	3	Cross-sectional	Jamar	Male	19.70	91.30	187	26.1	2.9	2.8	1.9	Central

Ten dance, TOP 6 in the world	Liiv et al., (2014) [83]	11	Cross-sectional	Holtain	Male	19.40	72.3	180.0	22.3	2.6	3.9	3.2	Ectomorphic mesomorph
Latin dance, TOP 6 in the world	Liiv et al., (2014) [83]	7	Cross-sectional	Holtain	Male	21.50	70.0	175.0	22.8	2.4	4.3	2.6	Balanced mesomorph
Ten dance, TOP 6 in the world	Liiv et al., (2014) [83]	11	Cross-sectional	Holtain	Female	19.00	55.5	167.0	19.9	3.1	3.0	3.4	Central
Latin dance, TOP 6 in the wolrd	Liiv et al., (2014) [83]	7	Cross-sectional	Holtain	Female	21.10	53.4	163.0	20.0	3.1	3.0	3.1	Central
Standard dance, TOP 6 in the world	Liiv et al., (2014) [83]	12	Cross-sectional	Holtain	Female	25.30	57.3	171.0	19.6	2.5	2.5	3.9	Balanced ectomorph
Standard dance, TOP 6 in the world	Liiv et al., (2014) [83]	12	Cross-sectional	Holtain	Male	26.70	72.5	183.0	21.6	2.2	3.7	3.6	Mesomorph-ectomorph
Olympic wrestling, National Institute of Sports, Physical Education and Recreation of Valle del Cauca, Colombia	Ramirez-Velez et al., (2014) [84]	21	Cross-sectional	Lange	Male	27.90	66.3	165.6	24.1	3.8	5.3	1.6	Endomorphic mesomorph
Rhythmic gymnastics, National Championships 2012, Serbia	Purenović-Ivanović & Popović, (2014) [85]	5	Cross-sectional	Not specified	Female	18.13	57.6	168.9	20.1	4.6	3.5	3.5	Balanced endomorph

Surf, International Wolrd Qualifying Series 5-star event 2010	Barlow et al., (2014) [86]	17	Cross-sectional	Harpenden	Male	34.12	78.57	177.2	25.0	2.48	5	1	Endomorphic mesomorph
Elite boxing, South Korea<br><br>Category: lightweight	Noh et al., (2014) [87]	6	Cross-sectional	Jamar	Male	19.3	58.3	168.1	20.6	1.8	3.2	3.1	Mesomorph-ectomorph
Elite boxing, South Korea<br><br>Category: light middleweight	Noh et al., (2014) [87]	8	Cross-sectional	Jamar	Male	19.3	68	171.4	23.2	2.3	3.7	2.2	Balanced mesomorph
Elite boxing, South Korea<br><br>Category: middleweight	Noh et al., (2014) [87]	5	Cross-sectional	Jamar	Male	19.3	72.5	177.6	23.1	2.1	3.5	2.6	Balanced mesomorph
Elite boxing, South Korea<br><br>Category: heavyweight	Noh et al., (2014) [87]	4	Cross-sectional	Jamar	Male	19.3	89.3	179	27.8	3.5	4.5	1.1	Endomorphic mesomorph
Jiu-Jitsu, World BJJ Championship 2012<br><br>Guard fighter	Báez et al., (2014) [88]	15	Cross-sectional	Slim Guide	Male	25.33	75.9	176.6	24.3	2.17	5.9	2.07	Balanced mesomorph
Jiu-Jitsu, World BJJ Championship 2012	Báez et al., (2014) [88]	10	Cross-sectional	Slim Guide	Male	27.77	75	170.8	25.7	2.3	7	1.26	Endomorphic mesomorph

Pass fighter													
Soccer, reserve players 1st division of Brazil<br><br>Position: forward	Fidelix et al., (2014) [89]	18	Cross-sectional	Cescorf	Male	16.17	70.2	175.8	22.7	2.5	4.7	2.6	Balanced mesomorph
Soccer, reserve players 1st division of Brazil<br><br>Position: defense	Fidelix et al., (2014) [89]	22	Cross-sectional	Cescorf	Male	16.09	69.9	177.6	22.1	2.7	4.2	3	Balanced mesomorph
Soccer, reserve players 1st division of Brazil<br><br>Position: goalkeeper	Fidelix et al., (2014) [89]	7	Cross-sectional	Cescorf	Male	16.29	80.5	188	22.7	3.1	4.1	2.9	Balanced mesomorph
Soccer, reserve players 1st division of Brazil<br><br>Position: midfielder	Fidelix et al., (2014) [89]	20	Cross-sectional	Cescorf	Male	16.4	68.6	175.9	22.1	2.3	4.1	2.9	Ectomorphic mesomorph
Elite judo, South Korea<br><br>Category: < 60 kg	Noh, et al., (2014) [90]	7	Cross-sectional	Jamar	Male	20.60	64.20	165.9	23.6	1.5	4.1	1.8	Balanced mesomorph
Elite judo, South Korea<br><br>Category: < 66 kg	Noh, et al., (2014) [90]	7	Cross-sectional	Jamar	Male	20.60	71.80	171.7	24.5	1.4	4.2	1.7	Balanced mesomorph

Elite judo, South Korea<br><br>Category: < 73 kg	Noh, et al., (2014) [90]	6	Cross-sectional	Jamar	Male	20.60	78.30	173.7	26.1	1.8	5.1	1.3	Balanced mesomorph
Elite judo, South Korea<br><br>Category: < 81 kg	Noh, et al., (2014) [90]	6	Cross-sectional	Jamar	Male	20.60	84.30	176.8	27.2	1.9	5.2	1.1	Endomorphic mesomorph
Elite judo, South Korea<br><br>Category: < 90 kg	Noh, et al., (2014) [90]	4	Cross-sectional	Jamar	Male	20.60	94.90	185.8	27.7	2.2	4.8	1.3	Endomorphic mesomorph
Elite judo, South Korea<br><br>Category: < 100 kg	Noh, et al., (2014) [90]	7	Cross-sectional	Jamar	Male	20.60	104.60	177.7	33.4	3.6	6.3	0.3	Endomorphic mesomorph
Elite judo, South Korea<br><br>Category: > 100 kg	Noh, et al., (2014) [90]	3	Cross-sectional	Jamar	Male	20.60	132.20	189	37.0	5	6.3	0.1	Endomorphic mesomorph
Basque pelota, Basque Ball Federation of Alava, U-23<br><br>Modality: individual	Urdampilleta et al., (2015) [91]	10	Cross-sectional	Holtain	Male	22.80	80.2	177.5	25.4	4.9	5.8	1.6	Endomorphic mesomorph
Basque pelota, Spanish professional teams	Urdampilleta et al., (2015) [91]	8	Cross-sectional	Holtain	Male	25.30	85.9	183.0	25.6	4.0	5.6	1.8	Endomorphic mesomorph

Modality: individual													
Tennis, University National Championship	Martinez-Rodriguez et al., (2015) [92]	26	Cross-sectional	Holtain	Male	23.00	74.7	180.0	23.0	3.3	4.3	2.5	Endomorphic mesomorph
Padel, University National Championship	Martinez-Rodriguez et al., (2015) [92]	21	Cross-sectional	Holtain	Male	23.10	74.3	180.0	22.9	3.7	4.1	2.4	Mesomorph-endomorph
Triathlon, championship of Spain university triathlon	Guillén et al., (2015) [93]	39	Cross-sectional	Holtain	Male	24.00	70.7	177.0	22.5	2.2	3.6	2.8	Ectomorphic mesomorph
Water polo, Spanish Honour Division<br><br>Position: center	Martínez et al., (2015) [94]	17	Cross-sectional	Not specified	Female	22	67.6	171.6	22.9	3.9	4.3	2.3	Mesomorph-endomorph
Water polo, Spanish Honour Division<br><br>Position: wing	Martínez et al., (2015) [94]	21	Cross-sectional	Not specified	Female	21.6	63.6	167.2	22.7	4.1	4.5	2.3	Mesomorph-endomorph
Water polo, Spanish Honour Division<br><br>Position: goalkeeper	Martínez et al., (2015) [94]	8	Cross-sectional	Not specified	Female	25.9	71	177	22.6	4.1	3.7	2.7	Mesomorph-endomorph
Judo, Spanish National Judo Team	Casals et al., (2015) [95]	9	Cross-sectional	Holtain	Male	20.00	85.9	180.0	26.5	1.9	5.6	1.6	Balanced mesomorph

Judo, Spanish National Judo Team	Casals et al., (2015) [95]	17	Cross-sectional	Holtain	Female	20.00	62.4	164.0	23.2	2.8	4.4	1.7	Endomorphic mesomorph
Baseball, Spanish Honour Division<br><br>Position: pitcher	Clavijo-Redondo et al., (2016) [96]	Not specified	Cross-sectional	Holtain	Male	23.87	83.63	179.33	26.1	5.52	4.62	1.5	Mesomorphic endomorph
Baseball, Spanish Honour Division<br><br>Position: catcher	Clavijo-Redondo et al., (2016) [96]	Not specified	Cross-sectional	Holtain	Male	23.87	83.43	182.24	25.2	5.62	5.01	2.04	Mesomorphic endomorph
Baseball, Spanish Honour Division<br><br>Position: first baseman	Clavijo-Redondo et al., (2016) [96]	Not specified	Cross-sectional	Holtain	Male	23.87	84.82	182.41	25.6	5.15	5.38	1.92	Mesomorph-endomorph
Baseball, Spanish Honour Division<br><br>Position: second baseman	Clavijo-Redondo et al., (2016) [96]	Not specified	Cross-sectional	Holtain	Male	23.87	85.33	181.01	26.0	5.58	4.37	1.63	Mesomorphic endomorph
Baseball, Spanish Honour Division<br><br>Position: third baseman	Clavijo-Redondo et al., (2016) [96]	Not specified	Cross-sectional	Holtain	Male	23.87	85.92	182.3	25.9	5.38	5.09	1.79	Mesomorph-endomorph
Baseball, Spanish Honour Division<br><br>Position: shortstop	Clavijo-Redondo et al., (2016) [96]	Not specified	Cross-sectional	Holtain	Male	23.87	84.43	182.89	25.5	5.27	5.39	2.05	Mesomorph-endomorph
Baseball, Spanish Honour Division	Clavijo-Redondo et al., (2016) [96]	Not specified	Cross-sectional	Holtain	Male	23.87	85.52	181.49	26.1	5.46	4.08	1.66	Mesomorphic endomorph

Position: left fielder													
Baseball, Spanish Honour Division<br><br>Position: central fielder	Clavijo-Redondo et al., (2016) [96]	Not specified	Cross-sectional	Holtain	Male	23.87	83.97	181.98	25.6	5.64	5.17	1.94	Mesomorph-endomorph
Baseball, Spanish Honour Division<br><br>Position: right fielder	Clavijo-Redondo et al., (2016) [96]	Not specified	Cross-sectional	Holtain	Male	23.87	85.23	182.45	25.7	5.54	4.7	1.88	Mesomorphic endomorph
Rhythmic gymnastics, Chile's National Team	Arriaza et al., (2016) [97]	21	Cross-sectional	Harpenden	Female	17.00	52.06	160.87	20.3	2.9	3.4	3	Central
Mixed martial arts	Marinho et al., (2016) [98]	8	Cross-sectional	Cescorf	Male	31.00	82.10	177	26.2	2.9	6.4	1.9	Endomorphic mesomorph
Sprint (100 m), TOP athletes from Italy and Croatia	Barbieri et al., (2017) [99]	98	Cross-sectional	Lange	Male	23.10	73.00	177.9	23.3	2.1	5	2.6	Balanced mesomorph
Judo, 10 years of experience	Buśko et al., (2017) [100]	15	Cross-sectional	Harpenden	Male	18.60	80.29	177.37	25.6	3.2	5.87	1.83	Endomorphic mesomorph
Soccer, 1st division of Mexico	Zuñiga et al., (2018) [101]	18	Cross-sectional	Slim Guide	Male	25.8	75.1	175.1	24.5	2.3	5.7	1.9	Balanced mesomorph
Racewalking Pan American Games	Díaz et al., (2018) [102]	10	Cross-sectional	Slim Guide	Male	17.20	60.32	171.2	20.6	2.34	3.33	3.39	Mesomorph-ectomorph

Racewalking Pan American Games	Díaz et al., (2018) [102]	20	Cross-sectional	Slim Guide	Female	19.45	55.34	163.6	20.8	3.11	3.04	2.93	Central
Basketball, Poland national team.	Gryko et al., (2018) [103]	35	Cross-sectional	Harpenden	Male	24.45	90.2	193.4	24.1	2.26	4.57	3.04	Ectomorphic mesomorph
Mountain biking, World Championship, 2011 UCI World Cup	Sánchez-Muñoz et al., (2018) [104]	22	Cross-sectional	Holtain	Male	28.00	67.10	175.5	21.9	1.7	4.6	3.1	Ectomorphic mesomorph
Mountain biking, 2011 UCI XCO Mountain Bike World Cup	Sánchez-Muñoz et al., (2018) [104]	5	Cross-sectional	Holtain	Male	30.30	70.20	178.4	22.1	1.8	4.8	3.1	Ectomorphic mesomorph
Long-distance runners, elite of Brazil<br><br>(military athletes)	Xavier et al., (2019) [105]	17	Cross-sectional	Lange	Male	23.70	65.30	175.5	21.3	1.8	4.2	3.3	Ectomorphic mesomorph
Judo, black belt medalists	Quintero et al., (2019) [106]	8	Cross-sectional	Cescorf	Female	21.00	61.00	157.7	24.7	4.94	5.6	1.3	Endomorphic mesomorph
Judo, black belt medalists	Quintero et al., (2019) [106]	7	Cross-sectional	Cescorf	Male	20.50	73.10	171.6	24.9	3.5	6.24	1.5	Endomorphic mesomorph
Futsal, 20th Brazilian Women’s Futsal Cup	Ferreira et al., (2020) [107]	115	Case-control	Cescorf	Female	22.00	58.60	161.8	22.6	4.5	4.1	2	Mesomorph-endomorph
Ballet of the Professional Conservatory of Granada, Spain	Alvero-Cruz et al., (2020) [108]	49	Cross-sectional	Holtain	Female	15.37	52.3	159	20.7	3.19	5.17	2.62	Endomorphic mesomorph

Soccer, 1st division of Italy	Campa et al., (2020) [19]	117	Cross-sectional	Lange	Male	13.50	53.50	162.3	20.4	2.1	4.1	3.3	Ectomorphic mesomorph
Judo, master belt (1-5 dan)<br><br>Categoría: < 60 kg	Roklicer et al., (2020) [109]	2	Cross-sectional	John Bull	Male	23.20	60.00	169.5	21.0	1.69	3.65	3.99	Mesomorph-ectomorph
Judo, master belt (1-5 dan)<br><br>Category: < 66 kg	Roklicer et al., (2020) [109]	13	Cross-sectional	John Bull	Male	23.20	66.00	175.38	21.5	2.01	3.7	3.62	Mesomorph-ectomorph
Judo, master belt (1-5 dan)<br><br>Category: < 73 kg	Roklicer et al., (2020) [109]	14	Cross-sectional	John Bull	Male	23.20	73.00	177.1	23.3	2.08	4.02	3.09	Ectomorphic mesomorph
Judo, master belt (1-5 dan)<br><br>Category: < 81 kg	Roklicer et al., (2020) [109]	11	Cross-sectional	John Bull	Male	23.20	81.00	179.09	25.3	2.23	4.89	2.49	Balanced mesomorph
Judo, master belt (1-5 dan)<br><br>Category: < 100 kg	Roklicer et al., (2020) [109]	15	Cross-sectional	John Bull	Male	23.20	90.00	183.87	26.9	2.33	5.5	1.93	Balanced mesomorph
Judo, master belt (1-5 dan)<br><br>Category: > 100 kg	Roklicer et al., (2020) [109]	4	Cross-sectional	John Bull	Male	23.20	100.00	190.88	27.7	2.62	5.34	2.04	Endomorphic mesomorph
Judo, master belt (1-5 dan)<br><br>Category: < 48 kg	Roklicer et al., (2020) [109]	2	Cross-sectional	John Bull	Male	23.20	100.00	198.75	25.5	2.92	6.35	0.91	Endomorphic mesomorph

Judo, master belt (1-5 dan)<br><br>Category: < 52 kg	Roklicer et al., (2020) [109]	2	Cross-sectional	John Bull	Female	22.30	48.00	146.5	22.5	2.17	5.11	1.4	Endomorphic mesomorph
Judo, master belt (1-5 dan)<br><br>Category: < 57 kg	Roklicer et al., (2020) [109]	2	Cross-sectional	John Bull	Female	22.30	52.00	161	20.0	2.2	2.71	3.48	Balanced ectomorph
Judo, master belt (1-5 dan)<br><br>Category: < 63 kg	Roklicer et al., (2020) [109]	9	Cross-sectional	John Bull	Female	22.30	57.00	162.6	21.7	2.8	3.62	2.73	Central
Judo, master belt (1-5 dan)<br><br>Category: < 70 kg	Roklicer et al., (2020) [109]	8	Cross-sectional	John Bull	Female	22.30	63.00	165.06	23.1	2.71	3.39	2.32	Balanced mesomorph
Judo, master belt (1-5 dan)<br><br>Category: < 78 kg	Roklicer et al., (2020) [109]	4	Cross-sectional	John Bull	Female	22.30	70.00	168.63	24.8	3.69	4.58	1.77	Endomorphic mesomorph
Judo, master belt (1-5 dan)<br><br>Category: > 78 kg	Mirali et al., (2021) [110]	5	Cross-sectional	John Bull	Female	22.30	78.00	170.2	26.9	3.07	4.99	1.21	Endomorphic mesomorph
Taekwondo, national and international ranking<br><br>Category: < 58 kg	Mirali et al., (2021) [110]	8	Cross-sectional	Slim Guide	Male	19.14	60.10	178.2	18.	1.3	2.9	4.8	Mesomorphic ectomorph
Taekwondo, national and international ranking	Mirali et al., (2021) [110]	8	Cross-sectional	Slim Guide	Male	20.20	56.40	174.8	18.6	1.2	3.2	4.8	Mesomorphic ectomorph

Category: < 54 kg													
Taekwondo, national and international ranking	Mirali et al., (2021) [110]	8	Cross-sectional	Slim Guide	Male	21.19	64.90	178.5	20.5	1.5	4.2	3.9	Mesomorph-ectomorph
Category: < 63 kg													
Taekwondo, national and international ranking	Mirali et al., (2021) [110]	7	Cross-sectional	Slim Guide	Male	21.24	82.50	187.7	23.6	2.1	4.6	3	Ectomorphic mesomorph
Category: < 80 kg													
Taekwondo, national and international ranking	Mirali et al., (2021) [110]	14	Cross-sectional	Slim Guide	Male	22.42	71.00	181.9	21.6	1.6	4.4	3.6	Mesomorphic ectotomorph
Category: < 68 kg													
Taekwondo, national and international ranking	Mirali et al., (2021) [110]	9	Cross-sectional	Slim Guide	Male	22.83	77.00	183.6	22.9	1.9	4.8	3	Mesomorphic ectotomorph
Category: < 74 kg													
Taekwondo, national and international ranking	Mirali et al., (2021) [110]	5	Cross-sectional	Slim Guide	Hombre	27.18	91.30	187.9	26.1	1.8	5.9	2	Balanced mesomorph
Category: > 87 kg													

Soccer, 1st division of Colombia<br><br>Position: goalkeeper	Castro et al., (2021) [111]	2	Cross-sectional	Harpenden	Male	21.00	80.90	185.2	23.6	1.7	5.8	2.8	Ectomorphic mesomorph
Soccer, 1st division of Colombia<br><br>Position: defender	Castro et al., (2021) [111]	5	Cross-sectional	Harpenden	Male	21.00	75.30	181.7	22.9	2	5	2.78	Ectomorphic mesomorph
Soccer, 1st division of Colombia<br><br>Position: midfielder	Castro et al., (2021) [111]	13	Cross-sectional	Harpenden	Male	21.00	69.70	175	22.7	2.1	5.3	2.7	Ectomorphic mesomorph
Soccer, 1st division of Colombia<br><br>Position: forward	Castro et al., (2021) [111]	4	Cross-sectional	Harpenden	Male	21.00	76.40	174.3	25.5	2.3	6.8	1.58	Endomorphic mesomorph
Rowing, Spanish National Team	Penichet-Tomas et al., (2021) [112]	13	Cross-sectional	Holtain	Male	27.30	75.30	182.1	22.7	1.8	4.5	3	Ectomorphic mesomorph
Rowing, Spanish National Team	Penichet-Tomas et al., (2021) [112]	11	Cross-sectional	Holtain	Female	27.70	61.90	169.9	21.6	2.9	3	2.9	Central
Soccer, 1st division of Chile<br><br>Position: goalkeeper	Hernández-Mosqueira et al., (2022) [113]	20	Cross-sectional	Slim Guide	Male	22.8	79	180.8	24.3	2.6	4.8	2.2	Balanced mesomorph

Soccer, 1st division of Chile<br><br>Position: defender	Hernández-Mosqueira et al., (2022) [113]	46	Cross-sectional	Slim Guide	Male	25.3	77.9	177.9	24.8	2.2	5.2	2.1	Balanced mesomorph
Soccer, 1st division of Chile<br><br>Position: midfielder	Hernández-Mosqueira et al., (2022) [113]	58	Cross-sectional	Slim Guide	Male	24.5	70.5	170.8	24.4	2.8	5.2	2	Endomorphic mesomorph
Soccer, 1st division of Chile<br><br>Position: forward	Hernández-Mosqueira et al., (2022) [113]	34	Cross-sectional	Slim Guide	Male	23.9	76.5	178.3	24.1	2.3	4.6	3.1	Ectomorphic mesomorph
Futsal, 1st division of Spain<br><br>Position: pivot wing	Castillo et al., (2022) [114]	28	Cross-sectional	Holtain	Female	22.70	60.36	168	21.4	4.01	3.17	2.03	Endomorphic mesomorph
Futsal, 1st division of Spain<br><br>Position: pivot	Castillo et al., (2022) [114]	7	Cross-sectional	Holtain	Female	25.00	65.43	166	23.7	4.28	4.13	1.6	Mesomorph-endomorph
Futsal, 1st division of Spain<br><br>Position: wing	Castillo et al., (2022) [114]	17	Cross-sectional	Holtain	Female	23.25	55.65	162	21.2	3.96	2.97	2.06	Mesomorphic endomorph
Futsal, 1st division of Spain<br><br>Position: forward	Castillo et al., (2022) [114]	20	Cross-sectional	Holtain	Female	26.89	58.63	163	22.0	3.78	3.67	1.92	Mesomorph-endomorph
Futsal, 1st division of Spain	Castillo et al., (2022) [114]	16	Cross-sectional	Holtain	Female	24.00	64.31	170	22.2	4.15	3.06	2.17	Mesomorphic endomorph

Position: goalkeeper													
Middle-distance runners (800 m, 1500 m), national competitions	Stachoń et al., (2023) [115]	22	Cross-sectional	GPM	Male	20.31	69.20	181.3	21.1	1.6	3.82	3.81	Mesomorph-ectomorph
Sprint (200 m, 400 m), national competitions	Stachoń et al., (2023) [115]	26	Cross-sectional	GPM	Male	20.37	74.30	180.6	22.9	1.68	4.94	2.9	Ectomorphic mesomorph
Long-distance runners (3000 m, 5000 m, 10000 m), national competitions	Stachoń et al., (2023) [115]	20	Cross-sectional	GPM	Male	21.39	67.60	177.3	21.5	2.11	4.72	3.36	Ectomorphic mesomorph
CrossFit® practitioners, national competitions	Cebrián-Ponce et al., (2024) [116]	107	Cross-sectional	Cescorf	Male	30.7	81.9	177.3	26.0	3.5	5.2	1.7	Endomorphic mesomorph
CrossFit® practitioners, national competitions	Cebrián-Ponce et al., (2024) [116]	38	Cross-sectional	Cescorf	Female	28.1	65.6	166.2	23.6	4.4	4.5	1.8	Mesomorph-endomorph
Traditional rowers from the first division of the traditional rowing league.<br><br>Position: Bow/Stern	Castañeda-Babarro et al., (2024) [117]	11	Cross-sectional	Holtain	Male	29.4	72.3	177.5	22.9	2.1	5.1	2.6	Balanced mesomorph
Traditional rowers from the first division of	Castañeda-Babarro et al., (2024) [117]	9	Cross-sectional	Holtain	Male	29.4	85.4	186.6	24.6	2.8	5.2	2.3	Balanced mesomorph

the traditional rowing league.<br><br>Position: 3rd/4th													
Basketball, Euroleague	Díaz-Martínez et al., (2024) [118]	22	Cross-sectional	Holtain	Male	23.42	96.9	196.9	25.2	2.84	4.72	2.88	Balanced mesomorph
Elite breakdancing, Italy	Ruscello et al., (2024) [119]	24	Cross-sectional	Holtain	Male	24.20	63.80	171	21.8	2.28	4.64	2.69	Balanced mesomorph
Elite breakdancing, Italy	Ruscello et al., (2024) [119]	9	Cross-sectional	Holtain	Female	21.90	54.20	158	21.7	2.34	5.16	2.38	Balanced mesomorph

BJJ: Brazilian jiu-jitsu; UCI: Union Cycliste Internationale; UCI World Cub XCO: Cross-Country Olympic Mountain bike race series; BMI: body mass index; ENDO: endomorphy; MESO: mesomorphy; ECTO: ectomorphy.
