# Supplementary material for: The Shape of Success: A Scoping Review of Somatotype in Modern Elite Athletes Across Various Sports
Source: Sports (Basel). 2025 Feb 4;13(2):38. doi: 10.3390/sports13020038 (PMC11860359; doi:10.3390/sports13020038)
Supplement: Supplementary file 1 [file sports-13-00038-s001.zip › Somatotype Table S3_SOMAREF_male athletes.pdf]

Table S3. References of somatotypes from male elite athletes (SomaRef).

| TEAM SPORTS | | | |
|---|---|---|---|
| **SPORT** | **ENDO** | **MESO** | **ECTO** |
| Baseball, first baseman | 3.38 | 6.83 | 0.6 |
| Baseball, infielder | 2.86 | 5.91 | 1.54 |
| Baseball, catcher | 4.08 | 6.84 | 0.32 |
| Baseball, outfielder | 2.96 | 6.66 | 0.66 |
| Baseball, pitcher | 3.26 | 5.66 | 1.47 |
| Basketball, forward | 2.8 | 3.7 | 3.8 |
| Basketball, guard | 3.2 | 4.2 | 3.2 |
| Basketball, center | 3.0 | 3.5 | 3.7 |
| Rugby, back | 2.2 | 6.5 | 1.2 |
| Rugby, forward | 2.8 | 7.2 | 0.8 |
| Volleyball, setter | 2.6 | 1.9 | 5.3 |
| Volleyball, opposite | 2.3 | 2.5 | 5.1 |
| Volleyball, center | 2.2 | 3.9 | 3.6 |
| Volleyball, hitter | 2.4 | 2.6 | 4.6 |
| Soccer, goalkeeper | 2.8 | 4.6 | 2.5 |
| Soccer, midfielder | 2.5 | 4.9 | 2.2 |
| Soccer, defender | 2.3 | 4.8 | 2.3 |
| Soccer, forward | 2.7 | 4.6 | 2.5 |
| Tennis | 2.9 | 4.6 | 2.5 |
| Water polo, wing | 2.53 | 5.01 | 2.4 |
| Water polo, center back | 3.0 | 5.8 | 1.9 |
| Water polo, center forward | 3.54 | 6.14 | 1.56 |
| **SPEED AND ENDURANCE SPORTS** | | | |
| **SPORT** | **ENDO** | **MESO** | **ECTO** |
| Marathon | 1.53 | 1.61 | 3.86 |
| Racewalking | 2.34 | 3.33 | 3.39 |
| Runners, (800 m, 1,500 m) | 1.6 | 3.82 | 3.81 |
| Runners, (3,000 m, 5,000 m, 10,000 m) | 2.11 | 4.72 | 3.36 |
| Sprint (100 m) | 2.1 | 5.0 | 2.6 |
| Sprint (200 m, 400 m) | 1.7 | 4.9 | 2.9 |
| Triathlon | 2.0 | 3.8 | 2.8 |
| **INDIVIDUAL SPORTS** | | | |
| **SPORT** | **ENDO** | **MESO** | **ECTO** |
| Gymnastics | 1.9 | 6.7 | 1.6 |
| Powerlifting, heavyweight | 6.3 | 10.7 | 0.1 |
| Powerlifting, lightweight | 3.2 | 7.5 | 1.1 |
| Powerlifting, middleweight | 3.2 | 8.0 | 0.7 |
| Windsurf | 2.3 | 5.0 | 2.4 |

| | | | |
|---|---|---|---|
| Sprint paddling | 2.6 | 4.6 | 3.1 |
| Mountain climbing | 1.55 | 5.28 | 2.64 |
| Surf | 2.48 | 5.0 | 1.0 |
| Tennis | 3.3 | 4.3 | 2.5 |
| Padel | 3.7 | 4.1 | 2.4 |
| Basque pelota | 4.5 | 5.7 | 1.7 |
| Mountain biking | 1.7 | 4.6 | 3.1 |
| Rowing, lightweight | 1.4 | 4.5 | 3.5 |
| Traditional rowing, bow/stern | 2.1 | 5.1 | 2.6 |
| Traditional rowing, 3rd/4th | 2.8 | 5.2 | 2.5 |
| CrossFit® practitioners | 3.5 | 5.2 | 1.7 |
| **COMBAT SPORTS** | | | |
| **SPORT** | **ENDO** | **MESO** | **ECTO** |
| Pencak silat | 3.2 | 5.6 | 2.0 |
| Greco-roman wrestling, heavyweight | 2.2 | 6.8 | 1.1 |
| Greco-roman wrestling, lightweight | 1.7 | 6.3 | 1.3 |
| Ssireum wrestiling, gyungjang | 3.2 | 5.6 | 1.3 |
| Ssireum wrestiling, sojang | 3.2 | 5.7 | 1 |
| Ssireum wrestiling, chungjang | 4.1 | 6.4 | 0.7 |
| Ssireum wrestiling, yongjang, | 4.2 | 5.7 | 0.6 |
| Ssireum wrestiling, yongsa | 4.5 | 5.9 | 0.6 |
| Ssireum wrestiling, yeoksa | 5.8 | 6.6 | 0.2 |
| Ssireum wrestiling, jangsa | 7.2 | 8.2 | 0.1 |
| Olympic wrestling | 3.8 | 5.3 | 1.6 |
| Boxing, lightweight | 1.8 | 3.2 | 3.1 |
| Boxing, light middleweight | 2.3 | 3.7 | 2.2 |
| Boxing, middleweight | 2.1 | 3.5 | 2.6 |
| Boxing, heavyweight | 3.5 | 4.5 | 1.1 |
| Jiu-jitsu pass fighter | 2.3 | 7.0 | 1.26 |
| Jiu-jitsu guard fighter | 2.17 | 5.9 | 2.07 |
| Mixed artial arts | 2.9 | 6.4 | 1.9 |
| Taekwondo, >80 kg | 2.9 | 2.8 | 1.9 |
| Taekwondo, <54 kg | 1.2 | 3.2 | 4.8 |
| Taekwondo, <63 kg | 1.5 | 4.2 | 3.9 |
| Taekwondo, <74 kg | 1.9 | 4.8 | 3.0 |
| Taekwondo, <87 kg | 1.8 | 5.9 | 2.0 |
| Taekwondo, <58 kg | 1.6 | 2.8 | 4.3 |
| Taekwondo, <68 kg | 1.9 | 3.4 | 3.5 |
| Taekwondo, <80 kg | 1.9 | 3.7 | 3.0 |
| Karate | 3.2 | 5.1 | 1.9 |

| | | | |
|---|---|---|---|
| Judo, >100 kg | 4.1 | 6.3 | 0.4 |
| Judo, <100 kg | 3.2 | 5.9 | 0.9 |
| Judo, <60 kg | 1.5 | 4.0 | 2.2 |
| Judo, <66 kg | 1.8 | 3.8 | 2.9 |
| Judo, <73 kg | 1.9 | 4.3 | 2.5 |
| Judo, <81 kg | 2.1 | 4.9 | 1.9 |
| **DANCE SPORTS** | | | |
| **SPORT** | **ENDO** | **MESO** | **ECTO** |
| Ten dance | 2.6 | 3.9 | 3.2 |
| Latin dance | 2.4 | 4.3 | 2.6 |
| Standard dance | 2.2 | 3.7 | 3.6 |
| Breakdancing | 2.28 | 4.64 | 2.69 |

*Note.* Table S2 presents reference values for somatotypes of modern elite athletes from the 'SomaRef' database, detailing the distribution of endomorphy, mesomorphy, and ectomorphy across various sports.
