# Supplementary material for: The Shape of Success: A Scoping Review of Somatotype in Modern Elite Athletes Across Various Sports
Source: Sports (Basel). 2025 Feb 4;13(2):38. doi: 10.3390/sports13020038 (PMC11860359; doi:10.3390/sports13020038)
Supplement: Supplementary file 1 [file sports-13-00038-s001.zip › Somatotype_Table S4_SOMAREF_female athletes.pdf]

Table S4. References of somatotypes from female elite athletes (SomaRef).

TEAM SPORTS			
**SPORT**	**ENDO**	**MESO**	**ECTO**
Soccer	3.1	3.6	2.4
Handball	4.2	4.7	1.8
Basketball	3.7	3.2	2.4
Water polo, center	3.9	4.3	2.3
Water polo, wing	4.1	4.5	2.3
Water polo, goalkeeper	4.1	3.7	2.7
Futsal, pivot wing	4.01	3.17	2.03
Futsal, pivot	4.28	4.13	1.6
Futsal, wing	3.96	2.97	2.06
Futsal, forward	3.78	3.67	1.92
Futsal, goalkeeper	4.15	3.06	2.17
Volleyball, hitter	2.9	3.2	3.0
Volleyball, center	2.9	2.9	3.3
Volleyball, setter	2.8	3.1	3.0
Volleyball, libero	3.3	4.0	2.3
Volleyball, opposite	3.0	3.4	2.9
**ENDURANCE SPORTS**			
**SPORT**	**ENDO**	**MESO**	**ECTO**
Triathlon	2.8	3.6	3.0
Racewalking	3.11	3.04	2.93
**COMBAT SPORTS**			
**SPORT**	**ENDO**	**MESO**	**ECTO**
Karate	3.1	3.7	2.4
Fencing	3.7	4.8	1.3
Pencak silat	4.0	4.1	1.9
Judo, <48 kg	2.17	5.11	1.4
Judo, <52 kg	2.2	2.71	3.48
Judo, <57 kg	2.8	3.62	2.73
Judo, <63 kg	2.71	3.39	2.32
Judo, <70 kg	3.69	4.58	1.77
Judo, <78 kg	3.07	4.99	1.21
Judo	4.9	5.6	1.7
**INDIVIDUAL SPORTS**			
**SPORT**	**ENDO**	**MESO**	**ECTO**
Bodybuilder	1.7	4.8	2.7
Sprint paddling	3.6	3.7	3.0
Rowing, lightweight	2.4	3.3	3.7
Gymnastics	3.2	3.4	3.0

CrossFit® practitioners	4.4	4.5	1.8
**DANCE SPORTS**			
**SPORT**	**ENDO**	**MESO**	**ECTO**
Ten dance	3.1	3.0	3.4
Latin dance	3.1	3.0	3.1
Standard dance	2.5	2.5	3.9
Ballet	3.19	5.17	2.62
Breakdancing	2.34	5.16	2.38

Note. Table S3 presents reference values for somatotypes of modern elite athletes from the 'SomaRef' database, detailing the distribution of endomorphy, mesomorphy, and ectomorphy across various sports.
