# Supplementary material for: The Shape of Success: A Scoping Review of Somatotype in Modern Elite Athletes Across Various Sports
Source: Sports (Basel). 2025 Feb 4;13(2):38. doi: 10.3390/sports13020038 (PMC11860359; doi:10.3390/sports13020038)

Figure S2. Somatoplots of female elite athletes

a) Team sports

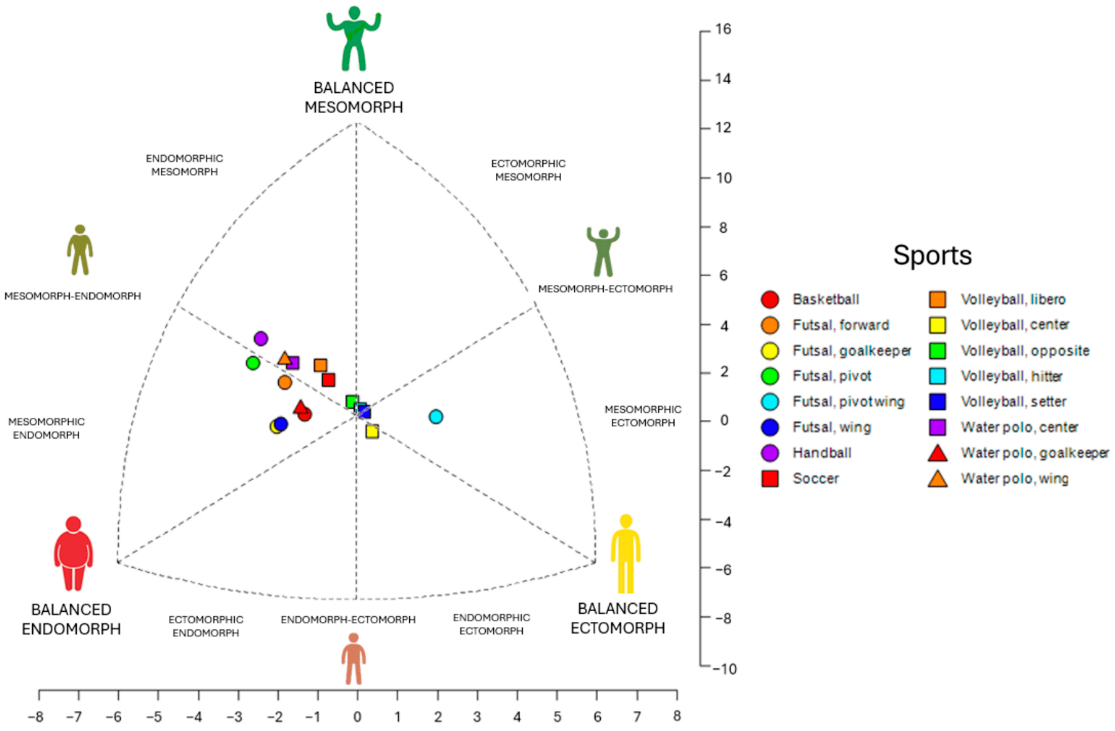

b) Combat sports

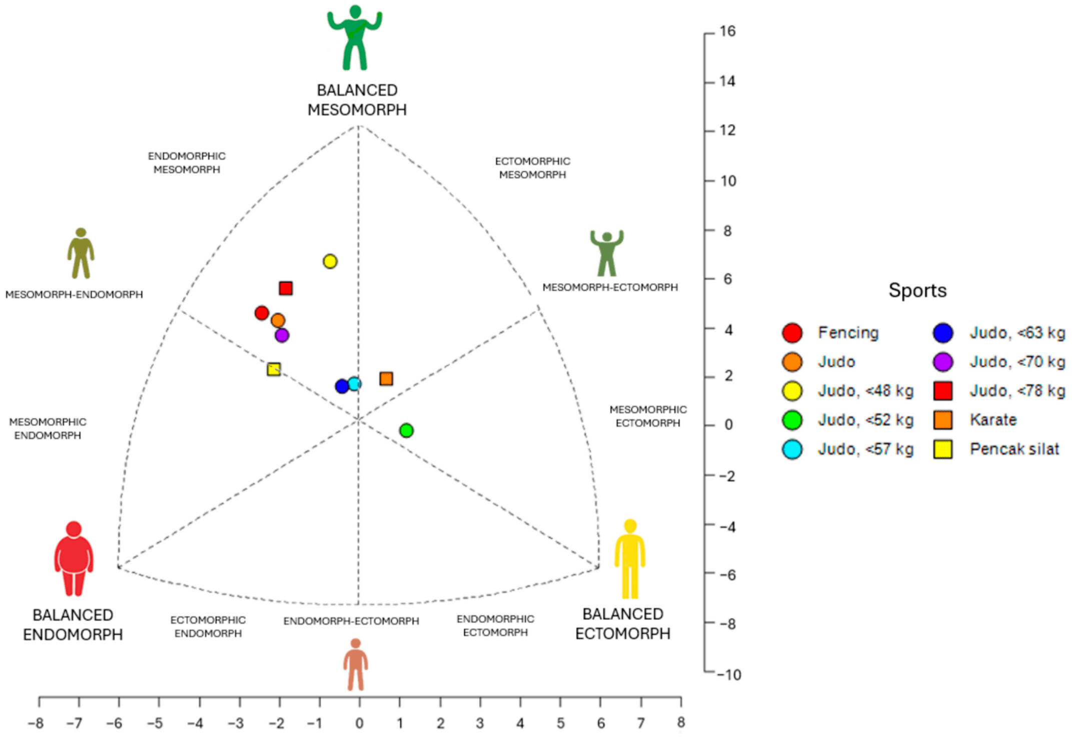

c) Endurance sports

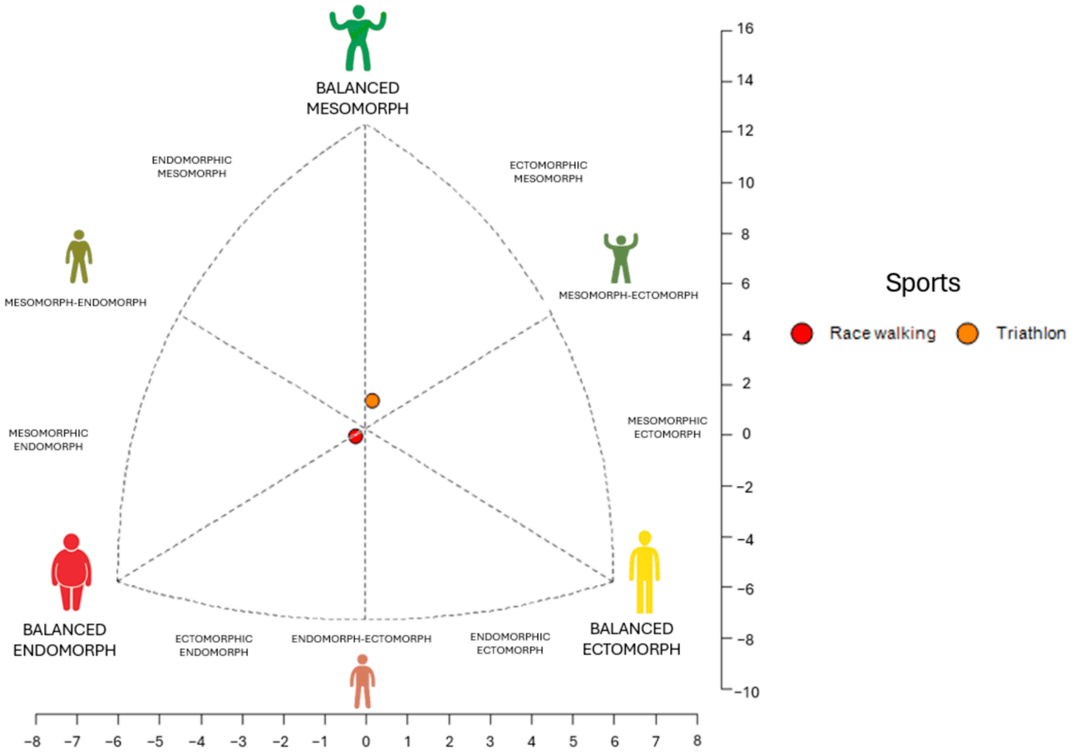

d) Individual sport

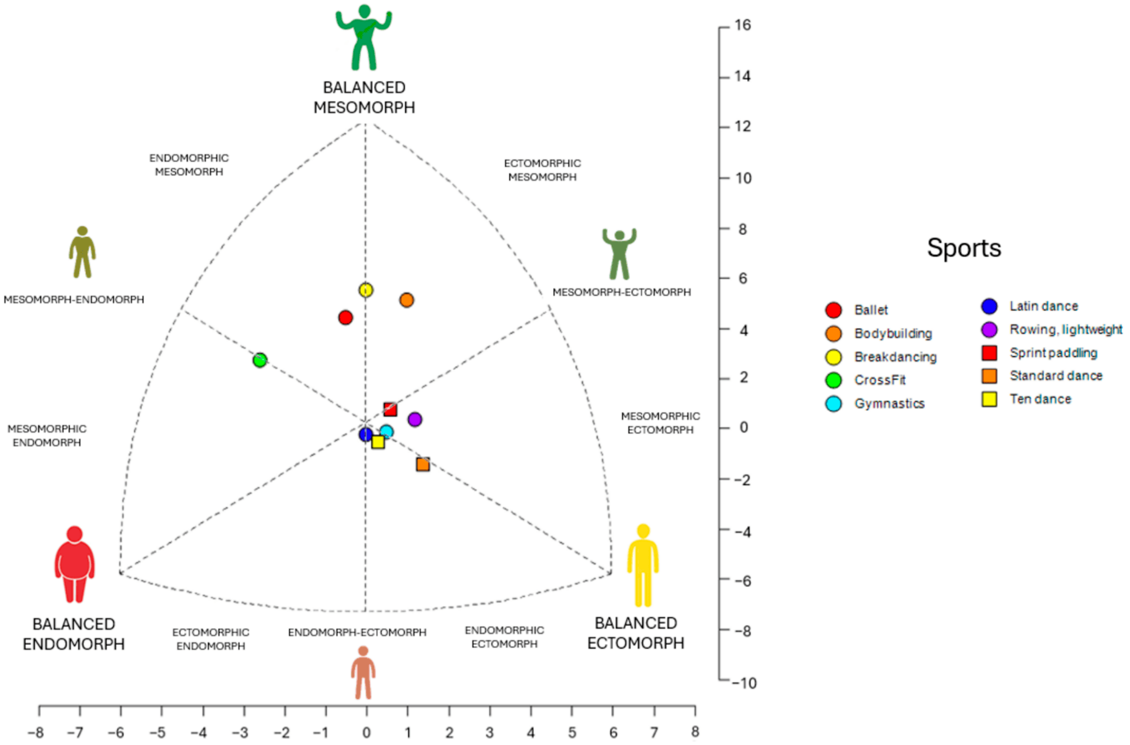

Supplement: Supplementary file 1 [file sports-13-00038-s001.zip › Somatoype Figure S2_female athletes.pdf]
